# Effects of Using Processed Amaranth Grain with and without Enzyme on Performance, Egg Quality, Antioxidant Status and Lipid Profile of Blood and Yolk Cholesterol in Laying Hens

**DOI:** 10.3390/ani12223123

**Published:** 2022-11-12

**Authors:** Babak Hosseintabar-Ghasemabad, Hossein Janmohammadi, Ali Hosseinkhani, Saeid Amirdahri, Payam Baghban-Kanani, Ivan Fedorovich Gorlov, Marina Ivanovna Slozhenkina, Alexander Anatolyevich Mosolov, Lourdes Suarez Ramirez, Alireza Seidavi

**Affiliations:** 1 Department of Animal Science, Faculty of Agriculture, University of Tabriz, Tabriz 5166616471, Iran; 2 Volga Region Research Institute of Manufacture and Processing of Meat-and-Milk Production, 400131 Volgograd, Russia; 3 Department of Animal Pathology, Animal Production, Bromatology and Food Technology, Veterinary Faculty, University of Las Palmas de Gran Canaria, 35412 Arucas, Spain; 4 Department of Animal Science, Rasht Branch, Islamic Azad University, Rasht 41335-3516, Iran

**Keywords:** amaranth grain, blood lipid, multienzyme, total antioxidant capacity, yolk cholesterol

## Abstract

**Simple Summary:**

Amaranth, as a valuable edible plant source, has a favorable potential to meet nutritional requirements for poultry. This research was conducted with the aim of investigating effects of feeding different levels of amaranth grain with and without enzyme on laying hens in order to evaluate performance, egg quality traits, antioxidant status and lipid profile of blood and yolk cholesterol. Based on the findings, feeding amaranth in laying hens can lead to production of low-cholesterol eggs without any negative effect on egg quality. Concomitantly, it improves the antioxidant status and atherogenic index (LDL/HDL) of the birds. In addition, feeding amaranth with enzyme additives led to improved performance in laying hens.

**Abstract:**

The objective of this study was to investigate the effects of feeding *Amaranthus hybridus chlorostachys* grain (AG) with (+E) and without enzyme (−E) on performance, egg quality, antioxidant status and lipid profile of blood serum and yolk cholesterol in laying hens. A total of 960 white leghorn (Hy-line W-36) commercial layers (56 weeks) were divided into 10 groups with 8 replicates per group (12 birds per replicate, including 3 adjacent cages with 4 birds each). A completely randomized design was implemented with a 5 × 2 factorial arrangement of treatments consisting of five levels of AG (0, 100, 200, 300 and 400 g/kg) and two levels of multienzyme complex addition (0 −E and 0.25 +E g/kg) fed to the hens for 12 weeks (2 wk. adaptation + 10 wk. main experiment). Feed intake (FI) and percentage of hen day production (HDP) were not affected by main effect of the AG level, but egg mass (EM) and egg weight (EW) were decreased (*p* < 0.01), and the feed conversion ratio (FCR) was impaired (*p* < 0.01). EM, EW and FCR were improved by enzyme addition (*p* < 0.01). EM, EW and FCR were affected (*p* < 0.01) by the interaction of AG and enzyme addition. The highest value of EM and the lowest value of FCR were observed in hens on the diet containing 200 g/kg AG with enzyme addition. Egg yolk cholesterol content was reduced (*p* < 0.05) by up to 10% with increasing levels AG in experimental diets. The egg quality traits, including Haugh units of protein quality, strength and shell thickness, were not affected by the main effects or interaction of AG and enzyme consumption. Amaranth feeding led to a decrease (*p* < 0.05) in triglyceride (TG) and low-density lipoprotein (LDL) while also promoting increases (*p* < 0.05) in the high-density lipoprotein (HDL) and total antioxidant capacity (TAC) of the blood. A comparison of the effects of contrasts showed that functional parameters (except FI), yolk cholesterol, antioxidant parameters (except MDA) and blood lipid profile had differed significantly (*p* < 0.05) between the hens fed amaranth versus those not fed amaranth. These findings indicate that feeding a diet containing up to 200 g/kg of AG with enzyme addition can improve EW, EM and FCR. Feeding laying hens diets containing AG also positively influenced blood traits and antioxidant status in laying hens while reducing egg yolk cholesterol content.

## 1. Introduction

Amaranth is considered one of the most important species on the neglected and underutilized species (NUS) checklist of edible plants according to the report of the World Health Organization (WHO, 2018, Geneva, Switzerland). That review reported that edible plants in the form of the NUS system are considered to be generative and within the economical safety net required to create food security and economic stability for farmers. These plants have high ecological compatibility, canbe produced at low cost and have many applications in the food and pharmaceutical industries. Additionally, this grain has potential for macro and retail development in the agricultural production sector, where many challenges exist [1,2,3,4]. Studies of amaranth use in human and animal nutrition began in the 20th century [5,6].

The Amaranth genus includes 75 species, among which 11 species grow in Iran [7]. Some amaranth species that can produce edible grain and that have extensive applications in nutrition (food) include *Amaranthus cruentus*, *A. caudatus*, *A. hybridus* and *A. hypochondriacus* [8]. *A. hypochondriacus* is a natural hybrid of *A. hybridus* × *A. caudatus* [6,9].

The protein content of amaranth grain is between 14 and 17%. In the protein value chart score published by the Food and Agriculture Organization (FAO, Rome, Italy), amaranth falls below egg and above cow’s milk. According to WHO reports, it is the second most valuable nutrient in the world and can supply the nutrient requirements recommended by the WHO for individuals [3,8,10,11]. Reports show that amaranth grain is gluten-free, and its lysine, methionine and arginine contents areabout 2–3 times greater than those of legumes (such as peas, beans, soybeans, etc.) and cereal grains (such as corn, wheat, rice, etc.) [3,12,13,14]. Alegbejo [15] reported that amaranth composition includes8% squalene, 2% tocopherols, 10% phospholipids and 2% phytosterols. In addition, a review of the scientific literature shows that amaranth grain contains non-nutritive compounds, including phenolics, saponins, tannins, phytic acid, oxalates, protease inhibitors, nitrates, non-starch polysaccharide, polyphenols and phytohemagglutinins [16,17,18,19];however, the amounts of non-nutritive components in amaranth are below values found in cereal grains [20,21,22].

The use of alternative cereal sources such as amaranth, which can maintain and improve poultry performance and have a positive effect on avian health, lead to additional benefits in laying hens, such as production of low-cholesterol eggs. Thus, amaranth is a promising option to improve food security and public health while reducing disease among consumers interested in low-cholesterol food sources [23]. A review of the literature shows that feeding amaranth can reduce blood lipid profiles, especially the blood cholesterol of mice, rabbits and poultry [24,25,26,27].

Punita and Chaturvedi [28] reported that feeding amaranth at a level of 250 g/kg in either raw or processed form to laying hens can effectively result in decreased triglyceride and yolk cholesterol content of the eggs laid, in addition to improving egg linoleic acid content. Popiela et al. [29] reported that feeding extruded amaranth grain to laying hens at a dose of 50 g/kg of body weightincreased performance and egg production, in addition to decreasing FCR. Rodríguez-Ríos et al. [30] evaluated amaranth consumption in laying hens and concluded that feeding amaranth at a dose of 150 g/kg feed did not negatively affect productive parameters and resulted in the production of low-cholesterol chicken eggs.

Owing to the presence of antinutrient components in amaranth, it is necessary to supplement diets with exogenous enzymes to reduce the negative effects of these components and simultaneously improve the efficiency of dietary energy and protein use in poultry [31].

We did not find any documentation on the characteristics and bioactive phytochemical composition of edible amaranth grain that is common in some countries, including Iran. On the other hand, previous studies have not reported the effects of feeding *Amaranthus hybridus chlorostachys* grain with enzymes included in the diets fed to laying hens. Thus, the objective of this study was to investigate the effects feeding amaranth (*A. hybridus chlorostachys*) grain, in the presence or absence of added enzyme, on productive performance and health of laying hens, including blood lipid profile parameters, antioxidant status and parameters related to production and quality of eggs.

## 2. Materials and Methods

All procedures and activities used in the present study related to the care, movement and sampling of birds were reviewed and approved by the Ethics, Care and use Committee of Animals at Tabriz University.

### 2.1. Animals and Diets

A total of 960 Hy-Line W-36 white leghorn laying hens (56 weeks of age) were used in a completely randomized design with a 5 × 2 factorial arrangement with amaranth grain (AG) and enzyme. The experiment included 10 experimental diets and 5 levels of AG (0, 100, 200, 300 and 400 g/kg) with and without enzyme addition (0 and 0.25 g/kg), and each diet included 8 replicates. Each replicate consisted of 12 hens that were housed in 3 separate cages with dimensions of 23 cm × 41 cm × 43 cm, and bird stocking density was based on hen welfare and cage size, with four birds in each cage. Each cage was fitted with water nipples and a galvanized feeding trough. The nipple drinkers (two nipples/cage) were positioned on the rear side of each cage. The hen house was provided with 16 h per day of light with a 20 lux intensity. The temperature of the hall was adjusted in the range of 18 to 22 °C. The same management conditions were maintained for all groups. The basal diet for the control group (i.e., fed 0 g/kg amaranth + 0 g/kg enzyme) was balanced according to nutrient recommended requirements of Hy-Line W-36 white leghorn laying hens, so a diet based on corn–soybean meal with apparent metabolizable energy corrected to zero nitrogen balance (AME_n_) 11.84 Mj/kg and crude protein (CP) 152.5 g/kg was considered.

Experimental diets comprised 5 levels of amaranth (0, 100, 200, 300 and 400 g/kg feed) and 2 levels of additive enzyme (0 and 0.25 g/kg feed). Assay diets were formulated on the basis of linear programming using UFFDA software and according to catalogue of leghorn hen requirements (Table 1).

Before starting the experiment, the egg production of the hens was recorded. Hens with approximately equal or similar egg production were grouped together in replications. The body weight (BW) of each laying hen was recorded at the beginning and the end of the experimental period. Additionally, the mortality rate was recorded during this period. The trials lasted 12 weeks, including 2 weeks of adaptation and 10 weeks of experimentation, and the results were reported at the end of the 10-week experimental period. In general, amounts of feed given each day, lighting program and temperature control were in accordance with the recommendations for Hy-Line W-36 white leghorn laying hens. Hens were provided ad libitum access to water for the duration of the trial and had free access to one of the ten experimental diets.

### 2.2. Test Ingredients

Amaranth grain, *A. hybridus chlorostachys* was provided by Darvash Giah Khazar medicinal herbs complex company (Ltf) in autoclaved form (Wet heat of 120 °C for 5 min). Crude protein (CP) content of AG was determined using a Foss 2300 Kjeletc analyzer, and ether extract (EE) by solvent extractor (Ser 148, Analytical Instrument Velp Scientifica) according to AOAC [32] was performed in the Advanced Animal Nutrition Laboratory of the University of Tabriz. Tocopherols were quantified by HPLC (model: Younglin Acme 9000, Anyang, Korea) with a Jasco FP-4025 detector (ABL & JASCO, Vienna, Austria) according to AOCS [33] methods.Phytosterol [34,35,36], linoleic acid (LA) and squalene values [37] in AG were measured by GC (model: Younglin 6100, Anyang, Korea) in the “specialized food laboratory Tekno Azma” in Tehran, an accredited laboratory approved by the Food and Drug Administration of Iran (Table 2). Profiles of amino acids, essential amino acids (EAA) and non-essential amino acids (NEAA) were determined in the laboratory of “Partov Bashash Danesh Gostar” located in Tehran, Iran, and the measurement protocol was based on the PicoTag method [38] using HPLC (Perkin-Elmer Co, Serie 200; Shelton, USA) (Table 2) [39].

The multienzyme preparation used in the present study, Natuzyme P_50_ (Bioproton Pty Ltf., Sunny bank, Queensland, Australia) contained cellulase (5,000,000 unit/kg), xylanase (10,000,000 unit/kg), pectinase (140,000 unit/kg), β-glucanase (1,000,000 unit/kg) with *Trichoderm areesei* and *Trichoderm alongibachiatum* origin (source) and α-amylase with *Bacillus subtilis* origin (source). It also contained protease (6,000,000 unit/kg) and phytase (500,000 unit/kg) from fungal origin of *Aspergillus niger*.

### 2.3. Performance

Feed intake (FI) was recorded as the amount of feed provided to each replicate. FI was weighed daily during the experiment, and feed consumption was measured weekly by subtracting the feed remaining from the quantity supplied to laying hens. Egg production was monitored daily and individually for each hen. The produced eggs were collected, counted and weighed for each replicate daily. Hen-day production (HDP) was calculated as the total number of eggs collected divided by the total number of live hens per day in each replicate. Egg mass (EM) was calculated per hen per day by multiplying the HDP by EW. The FCR was calculated as FI/EM.

### 2.4. Egg Quality Traits

The collected eggs were transferred to the Tabriz University Advanced Animal Nutrition Laboratory, where they were examined, and the egg quality traits were assessed 24 h after eggs were collected. Egg quality traits of shell thickness, shell strength, shape index and Haugh units of protein quality were recorded at the end of the experimental period (9 eggs from each replicate and 720 eggs in total).

Shell strength was measured using a digital eggshell force gauge (Wagner Instruments, Bridgeport, USA). Shell thickness was measured at three locations (the air cell, equatorand sharp end) using a digital micrometer instrument (Mitutoyo, Kawasaki, Japan). The yolk diameter (D) of all eggs was measured using a compass (Swordfish model, Tokyo, Japan), and the height yolk (HY) was measured using a tripod digital micrometer instrument (Mituoyo model, Kawasaki, Tokyo, Japan). Yolk index, Haugh units and egg specific gravity (ESG) were calculated based on the Formulae (1), (2) and (3), respectively. HY, HA, EW and SW represent height yolk, height albumin, egg weight and shell weight, respectively [40,41,42].
Yolk index = [HY/D] × 100Haugh unit =100 log HA + 7.57 − 1.7 EW ^0.37^ESG = EW ÷ (0.968 EW − 0.4759 SW)(Formula 1).(Formula 2).(Formula 3).

### 2.5. Cholesterol Content in Egg Yolk

At the end of the experimental feeding period, 6 eggs from each replicate (2 eggs per cage) for a total of 480 eggs (2 eggs × 3 cages × 8 replicates × 10 treatments), were transferred to Tabriz Medical Sciences Service Laboratory (Pashmineh) for measurement of cholesterol content in each egg yolk.

After separating the yolks from the egg albumen and weighing them with a glass mixer, the yolks were thoroughly stirred to obtain a homogeneoussample of one gram from each homogenized yolk, which was mixed with 50 mL sodium hydroxide, then neutralized with 50 mL hydrochloric acid. The samples were centrifuged at 25 °C for 10 min at 3000 rpm [43,44]. One ml of the sample solution was injected into an Microlab 300 autoanalyzer spectrophotometer (ELITech Group Company, Paris, France) using a commercial diagnostic laboratory kit. Cholesterol content (mg) was expressed as the amount of cholesterol per gram of yolk, and the amount of cholesterol was expressed as a proportion of the whole egg yolk (yolk weight × cholesterol per gram of yolk) [44,45].

### 2.6. Blood Biochemical Parameters

At the end of the experimental feeding period, six hens from each replicate (two from each cage) were selected randomly, and blood samples were taken from their wing vein and placed into additive-free blood tubes. The samples were transferred to the Pharmaceutical Analysis Research Center of Tabriz Medical University and centrifuged for ten minutes at 3000 rpm and 20 °C, and plasma was collected for later analysis. The plasma samples were stored at −85 °C in the lab for further analysis.

Plasma samples were separated to measure triglyceride content (TG), total cholesterol (TC), low-density lipoprotein (LDL)and high-density lipoprotein (HDL) using commercial diagnostic kits (enzyme method) [40,46].The TGs was measured using a colorimetric enzyme procedure, and TC, HDL and LDL were measured using enzymatic photometric methods [40,47]. The amounts were expressed as mg/dL unit, and the atherogenic index (AI) was calculated as the ratio of LDL/HDL [31,47].

### 2.7. Antioxidant Indices

At the time of blood collection, two milliliters of blood was transferred to a heparinized tubeand then placed inside a container of dry ice until the resulting plasma was collected according to the recommendations for analysis of the relevant blood antioxidants parameters [48].

The samples were analyzed for total antioxidant (TAC) and malondialdehyde (MDA) contents using a Randox kit according to the manufacturer’s recommendation (Germany). MDA was expressed as the level of peroxidation of blood plasma lipids, representing oxidative damage to a large extent. Therefore, using 1,1,3,3-tetra ethoxy propane to react with thiobarbituric acid (TBA) after extraction with isobutanol, the values of these two parameters were obtained using the methods described by Kei [49] and Yagi [50], and the optical absorbance of the solution was obtained by spectrophotometry (Shimadzu V-1201 model, Japan) at a wavelength of 532 nm [44].

In addition, the activities of aspartate aminotransferase (AST) and alanine aminotransferase (ALT) enzymes in the samples were determined using a spectrophotometer (Alycon 300 model, made in the United States) and a Pars Azmoon kit (made in Iran) following the method outlined by Baghban-Kanani et al. [41]. The expressions of TAC, AST and ALT were reported as micromole/mL, and MDA was reported as nanomol/mL.

### 2.8. Statistical Analysis

After collecting data and recording values in Excel 2013, in order to evaluate the correctness of the assumptions and check for random errors, the normality of residuals was first determined with the Shapiro–Wilk method and the homogeneity of variance of assay diets was determined using Bartlett’s test with “R” software [51]. Using the same software, all the data were statistically analyzed for a completely random design with a factorial arrangement of treatments, and mean values were reported as means ±SEM [52]. In order to determine significant differences among means, Duncan’s test was also used at the 5% level for type I errors [53]. The level of response to amaranth feeding was computed using orthogonal polynomial contrasts (linear and quadratic), and statistical significance was declared considered as *p* < 0.05. The statistical model of the completely random design with factorial arrangement of treatmentsfor the current research is as follows:

Y_ijk_ = µ + A_i_ + E_j_ + (A × S)_ij_+ e_ijk_

where Y_ijk_ = continuous variable, μ = total mean, A_i_ = the effect of amaranth on the diet (i contains 5 levels), E_j_ = the effect of enzymes (j contains 2 levels), (A × E)_ij_ = interaction of amaranth × enzymesand e_ijk_ = the error term of the experiment.

## 3. Results

The results presented in Table 3 show that the performance traits of laying hens, including EW, EM and FCR, were affected by AG consumption at a rate of 300 g/kg and 400 g/kg AG; EW and EM were decreased (*p* < 0.01), whereas FCR was increased (*p* < 0.01). However, FI and HDP were not affected. Enzyme addition increased (*p* < 0.01) the EW and EM of eggs and subsequently improved (*p* < 0.01) FCR.

For interaction of AG × enzyme, results show that feeding of amaranth up to 200 g/kg without enzyme (AG-E) did not have any negative effects on production performance and was similar to the control group (0 g/kg AG + no enzyme), but feeding AG to laying hens at 300 g/kg or 400 g/kg in the absence of enzyme led to a reduction (*p* < 0.01) in EW and EM. In addition, consumption of AGat levels higher than 100 g/kg in the absence of enzyme resulted in an increase in FCR (*p* < 0.01). Further examination of the interactions between experimental treatments indicates that the feeding amaranth plus enzyme (AG + E) to laying hens at 200 g/kg AG resulted in the highest (*p* < 0.01) EM and the lowest (*p* < 0.01) FCR.

A comparison of the contrasts of means showed that HDP, EW and EM differed significantly between hens fed no amaranth and those fed amaranth; the zero-amaranth group had the highest (*p* < 0.05) values. Additionally, the average values of FCR for amaranth-fed groups were highest, and hens fed zero amaranth improved (*p* < 0.05) FCR.

In addition, a linear regression equation was obtained for the HDP parameter (Y = 82.62 − 0.0074a), EW (Y = 60.49 − 0.0056a), EM (Y = 49.97 − 0.0090a) and FCR (Y = 2.18 − 0.0004a). The intercept of the linear equations show the extent to which the desired parameter decreased for each unit of increase in amaranth feeding (a). For functional parameters, only the quadratic equation for the HDP parameter was significant (*p* < 0.05).

Results for yolk cholesterol analysis are shown in Table 4. These results indicate that the main effect of amaranth feeding was a significant reduction in cholesterol in egg yolks; a 10% reduction was observed compared to hens fed the control diet. Feeding amaranth at 300 or 400 g/kg resulted in the greatest reduction (*p* < 0.05) in egg cholesterol. For other egg quality traits produced at the end of the period, including shell thickness and strength, shape indexand Haugh unit, no differences (*p* > 0.05) were observed between the experimental groups, indicating that feeding of amaranth with or without enzyme to laying hens has potential to reduce egg yolk cholesterol without negatively affecting egg quality traits.

A comparison of contrasts of means showed that the average egg yolk cholesterol of groups fed amaranth (11.54 or 199.63) was less (*p* < 0.05) than that of the experimental groups not fed amaranth (12.59 or 218.26). In addition, the linear regression equation was obtained for egg yolk cholesterol parameters (Y = 0.0030 − 12.36a) and (Y = 0.0566 − 214.70a), which showed that for each unit increase in amaranth feeding (a), yolk cholesterol decreased by 0.0030 and 0.0566 units, respectively.

Results of blood antioxidant status are shown in Table 5. The main effects of treatment indicated that amaranth consumption resulted in a significant increase (*p* < 0.05) in blood TAC of hens fed the experimental treatments. There was no difference (*p* > 0.05) in blood MDA of laying hens. Results showthat the feeding of 200 or 300 g/kg amaranth resulted in the lowest (*p* < 0.05) values of AST and ALT in the blood of the laying hens. The results for main effects of treatment and interaction of amaranth × enzyme on blood lipid profile showed that feeding hens diets containing amaranthled to a decrease (*p* < 0.05) in TG and LDL and a subsequent increase (*p* < 0.05) in blood HDL. However, feeding enzyme did not affect (*p* > 0.05) any of these parameters.

The AI (LDL/HDL) results showed that for main effects, feeding of 100, 200 or 300 g/kg of amaranth reduced (*p* < 0.05) AI. In contrast, feeding of enzyme increased (*p* < 0.05) AI.

Feeding amaranth, with or without enzyme addition, led to a reduction (*p* < 0.05) in AI, except when 400 g/kg amaranth with enzyme addition was fed. The contrasts of means showed that the difference in the average values of the two groups fed no amaranth (0 levels) differed (*p* < 0.05) from those fed amaranth for TAC, ALT, AST, TG, TC, LDL, HDL and AI parameters.

Linear regression equations were obtained for the TAC (Y = 6.30 + 0.0006a), ALT (Y = 5.11 − 0.0013a), AST (Y = 214.48 − 0.0051a), TG (Y = 95.02 − 0.0135a), TC (Y = 103.17 − 0.0214a), LDL (Y = 97.11 − 0.0072a), HDL (Y = 44.12 + 0.0220a) and AI (Y = 2.31 − 0.00009) parameters. The intercepts obtained in these equations show the amount of increase (+) or decrease (−) in the desired parameters occurred for each unit of increased amaranth (a) fed. The quadratic equations for TC, HDL and AI parameters were significant (*p* < 0.05).

## 4. Discussion

The phytochemical bioactive compounds found in amaranth grain fed to laying hens (CP, EAA and NEAA) in the present study are shown in Table 2. The relatively abundant values from phytosterols and tocopherols indicate the antioxidant capacity. Additionally, the presence of EAA, NEAA, linoleic acid and squalene found in amaranth grain demonstrates its nutritional value. Table 2 also shows that AG contains all the amino acids in balance for monogastric animals. Chemical compounds and apparent metabolizable energy corrected for nitrogen balance (AME_n_) were measured in amaranth and have been reported previously [54]. The concentration of compounds reported in the present study are in line with the reports of Tang and Tsao [27], Ogrodowska et al. [55], Iftikhar and Khan [56], Waisundara [57] and Tareh et al. [58], suggesting that amaranth grain is suitable for inclusion in livestock rations.

The use of extruded amaranth grain in the diet of laying hens was previously reported by Tillman and Waldroup [59], who they included this product at 100 and 200 g/kg. Similarly, Popiela et al. [29] fed amaranth to laying hens at 50 g/kg, which improved EW and FCR, consistent to the current findings. The significant increase EW can be attributed to increased amounts of linoleic acid and EAA in amaranth grain compared to other commonly fed cereals, attributes that are among the most important factors affecting the shape and EW of eggs. Owing to the nutrient balance of amaranth grain, heat processing and inclusion of a multienzyme additive may improve nutrient bioavailability and reduce antinutrient effects. The improvement in EW, along with maintainence of egg shell strength, in the present study may have resulted from the appropriate bioavailability of calcium and other elements needed to maintain eggshell structure [60]. Positive effects of feeding AG in the diets of laying hens on the performance and egg quality traits were observed in the present study. However, many edible plants, such as amaranth, contain antinutrient factors thatlimit their use as a feedstuff for livestock. Therefore, the inclusion of exogenous enzyme additives in diets that are of fungal and bacterial origin can reduce the effects of antinutritional factors naturallyoccurring in amaranth [61]. A literature review of enzyme preparations used in the poultry feed industry shows that supplementing poultry diets with multiple enzymes can improve the activity of lipase and chymotrypsin enzymes in the gastrointestinal tract through synergism with endogenous enzymes [31]. It can also reduce and degrade antinutrients, such as non-starch polysaccharides [62], and reduce viscosity [63] to increase the availability of important nutrients, such as starch, protein and minerals, within the cell walls of highcrude fiber to ultimately improve protein digestibility and metabolizable energy [40,64,65].Baghban-Kanani et al. [31], Alam et al. [66] and Rutherfurd et al. [67] reported that supplementing laying hen diets with enzyme additives resulted in increased egg weight due to reduced viscosity; separation of large molecules; and improved availability of starch, protein, fat and metabolizable energy, which is consistent with the findings of the present study.

The reduced yolk cholesterol of eggs from hens fed amaranth in the present study is consistent with previous reports by Punita and Chaturvedi [28], Rodríguez-Ríos et al. [30], Reklewska et al. [68], Bartkowiak et al. [69], Króliczewska et al. [70], Longato et al. [71] and Divari et al. [72], showing that yolk cholesterol was reduced in eggs from laying hens fed amaranth grains at levels of 50 to 300 g/kg. The mechanism of reduced egg cholesterol content (discussed subsequently) was positively correlated with reduced cholesterol in the blood and liver of hens [73]. On the other hand, Case et al. [74]. reported that 5% inhibition of HMC-CoA reductase activity leads to a 2% decrease in blood serum cholesterol. Those researchers believe that some known and specific compounds unique to amaranth can limit the rate of production of the liver enzyme 3-hydroxy-3-methylglutaryl coenzyme-A (HMG-CoA) reductase, resulting in reduced cholesterol. In general, many edible plants and even byproducts derived from such plants contain fiber, phenolic compounds and/or bioactive compounds that increase the cholesterol-lowering effects by binding to and excreting cholesterol while improving egg quality traits without adversely impacting performance and health [40,65,75]. The abundant linoleic acid (LA) in amaranth grains can play an important role in the excretion of bile acids and reduce cholesterol [41]. Bartkowiak et al. [69] concluded that feeding of 50 g/kg amaranth to hens can lead to production of eggs that are low in cholesterol and rich in linoleic acid. Amaranth grain also contains squalene, which, when fed to livestock, can be transported to the liver with the help of chylomicrons and be used to make steroids and bile acids. Bile acids (colic and deoxycholic) are synthesized from squalene in hepatic cells. In turn, bile acids can combine with glycine and taurine to produce bile salts and reduce yolk cholesterol by increasing bile secretion turnover, therefore reducing egg cholesterol content [31,40,41,44,65,75].

Positive effects of amaranth consumption by hens on the health parameters of blood, especially lipid profiles, including cholesterol, observed in the present study are consistent with previous findings. Qureshi et al. [24] reported that the reason for a 10to 30%-reduction in blood cholesterol of chickens fed diets containing amaranth grains was an 18 and 9%reduction in the enzymatic activity of cholesterol 7-alpha hydroxylase, 3-hydroxy-3-methylglutaryl coenzyme A reductase in the liver, which led to a significant reduction in blood cholesterol. Activation of the enzyme 7-alpha hydroxylase also leads to the formation of bile acids from cholesterol and increases catabolism and cholesterol excretion. Mendonça et al. [25] and Soares et al. [26] confirmed that the cholesterol-lowering effects of amaranth fed to hamsters was due to the presence of peptides that limit the production of HMG-CoA reductase. The analysis of tocopherol and fatty acids in the present study attributed those compounds to the process of reducing cholesterol, LDL and TG while improving blood HDL. These effects are consistent with reports by other researchers who believe that vitamin E isomers in amaranth grain oil have effects that can reduce cholesterol and LDL in blood by 30 and 70%, respectively [27,76,77]. The linoleic acid composition in amaranth was shown to have a significant effect on reducing LDL cholesterol and increasing blood HDL without causing side effects [27,76,77]. Janmohammadi et al. [78] also reported that the there was a synergistic effect of heat treatment and enzyme inclusion on the metabolisable energy concentration of amaranth in the diets of broilers. Longato et al. [71] and Peiretti et al. [17] reported that feeding of AG to broiler chickens led to a significant reduction triglycerides and cholesterol in blood. Szczerbinska et al. [79] studied the feeding of amaranth to Japanese quail (Coturnix coturnix japonica) and reported that amaranth at 70 g/kg in the diet of these birdssignificantly reduced cholesterol and blood triglycerides. Chaturvedi et al. [80] also reported that rats fed AG had exhibited a decrease in TG and a subsequent increase in HDL and that effects of hypocholesterolemia were significant. The results of the present study are consistent with previous findings.

Popiela et al. [29] showed that ALT and AST decreased five weeks after feeding 5 or 100 g/kg amaranth to laying hens. Similarly, Szczerbinska et al. [79] reported a significant decrease in ALT and AST when 70 g/kg amaranth was included in the diets of Japanese quail (*Coturnix coturnix japonica*). In contrast, Króliczewska et al. [70] reported that feeding of 20, 50 or 100 g/kg amaranth to laying hens led to a significant increase in ALT and AST activity, whereas Longato et al. [71] reported that feeding of amaranth to broilers did not affect the values of ALT. Zraly et al. [81] observed that including amaranth in the diet of pigs increased ALT but did not affect AST. However, in laboratory animals, the age of the animal for serum sampling to measure AST and ALT resulted in differences in serum antioxidant parameters of animals fed diets containing amaranth. Tsao and Tang [27] believe that the only active ingredient in amaranth grains is amaranthine and iso-amaranthine, which can increase the accumulation of tocopherol in serum and tissues and play an important role in improving the antioxidant activity in blood of animals. In general, however, the results with respect to improving the antioxidant parameters in birds consuming amaranth grains in many reports over the last decade [57] indicate the high health and healing power of this valuable grain, and the results of the present study are consistentwith those of previous studies.

## 5. Conclusions

Overall, the results of our study indicate that feeding amaranth grain was beneficial to the health parameters of laying hens, improving antioxidant status, as well as lowering cholesterol, LDL and TG while increasing blood HDL. It is worth mentioning that production and egg quality traits were improved when a multienzyme additive was included with amaranth grain fed up to 200 g/kg levels. The superior quality CP in amaranth grain compared to corn, as well as the presence of vitamin E and squalene, are important reasons for choosing this source in countries such as Iran, which is struggling with climate stress. Finally, egg yolk cholesterol often plays a fundamental role in consumer choices of food products. Of amaranth grains are used in the diet of laying hens, a reduction in yolk cholesterol of up to 10% can be expected, which is a promising factor for consumers focused on their dietary health.

## Figures and Tables

**Table 1:** Ingredients and chemical composition of experimental diets fed to laying hens.

Treatments ^1^: Amaranth × Enzyme										
Item	T_1_: 0 × 0	T_2_: 100 × 0	T_3_: 200 × 0	T_4_: 300 × 0	T_5_: 400 × 0	T_6_: 0 × 0.25	T_7_: 100 × 0.25	T_8_: 200 × 0.25	T_9_: 300 × 0.25	T_10_: 400 × 0.25
Ingredient, (g/kg)										
Corn	614.9	548.0	481.5	415.7	326.9	612.2	545.4	478.9	413.0	329.4
Soybean meal	235.6	217.0	198.2	178.6	162.0	236.5	217.8	199.1	179.5	162.0
Amaranth grain	0.0	100.0	200.0	300.0	400.0	0.0	100.0	200.0	300.0	400.0
Oyster shell	96.7	91.1	85.4	79.7	85.5	96.7	91.1	85.4	79.7	83.0
Vegetable oil	24.0	16.2	08.4	00.3	00.0	25.8	18.0	10.1	02.1	00.0
Dicalcium phosphate	18.8	18.8	18.8	18.7	18.6	18.8	18.8	18.8	18.7	18.6
Vitamin premix ^2^	2.5	2.5	2.5	2.5	2.5	2.5	2.5	2.5	2.5	2.5
Mineral premix ^3^	2.5	2.5	2.5	2.5	2.5	2.5	2.5	2.5	2.5	2.5
Common salt	2.0	2.0	2.0	2.0	2.0	2.0	2.0	2.0	2.0	2.0
DL-methionine	3.0	1.9	0.7	0.0	0.0	3.0	1.9	0.7	0.0	0.0
Natuzyme P_50_ enzyme	00.0	00.0	00.0	00.0	00.0	0.25	0.25	0.25	0.25	0.25
Calculated nutrient content, (g/kg)										
AME_n_ (Mj/kg)	11.84	11.84	11.84	11.84	11.84	11.84	11.84	11.84	11.84	11.84
Crude protein (g/kg)	152.5	152.5	152.5	152.5	152.5	152.5	152.5	152.5	152.5	152.5
Ether extract (g/kg)	50.2	46.4	42.5	38.5	41.4	51.8	48.0	44.1	38.7	41.4
Crude fiber (g/kg)	27.8	38.3	48.8	59.2	69.4	27.8	38.2	48.7	59.2	69.4
Linoleic acid (g/kg)	15.8	39.5	43.1	50.6	58.6	15.8	39.5	43.1	50.6	58.6
Calcium (g/kg)	43.5	43.5	43.5	43.5	43.5	43.5	43.5	43.5	43.5	43.5
Available phosphorus (g/kg)	4.6	4.6	4.6	4.6	4.6	4.6	4.6	4.6	4.6	4.6
Methionine (g/kg)	3.7	4.1	4.2	4.2	4.2	3.7	4.1	4.2	4.2	4.2
Met + cysteine (g/kg)	6.7	6.7	6.7	7.2	7.2	6.7	6.7	6.7	7.2	7.2
Lysine (g/kg)	0.78	0.79	0.81	0.81	0.82	0.78	0.79	0.81	0.81	0.82
Arginine (g/kg)	8.1	8.2	8.2	8.5	8.6	8.1	8.2	8.2	8.5	8.6
Threonine (g/kg)	5.7	5.8	5.9	6.1	6.1	5.7	5.8	5.9	6.1	6.1
DCAB (mEq/kg)	201.47	201.47	201.47	201.47	201.47	201.47	201.47	201.47	201.47	201.47

^1^**Treatments** (g/kg): **T_1_**: 0 AG(−E), **T_2_**: 100 AG(−E), **T_3_**: 200 AG(−E), **T_4_**: 300 AG(−E), **T_5_**: 400 AG(−E), **T_6_**: 0 AG(+E), **T_7_**: 100 AG(+E), **T_8_**: 200 AG(+E), **T_9_**: 300 AG(+E)**, T_10_**: 400 AG(+E); ^2^ vitamin supplement provided per kilogram of diet:vitamin A, 8000 IU; vitamin E, 20 IU; menadione, 3.0 mg; vitamin D3, 2000 IU; riboflavin, 4.0 mg; Ca-pantothenate, 12 mg; nicotinic acid, 50 mg; choline, 300 mg; vitamin B_12_, 15 mg; vitamin B_6_, 0.12 mg; thiamine, 1.5 mg; folic acid, 1.00 mg; D-biotin, 0.10 mg; ^3^ mineral supplement provided per kilogram of diet: trace mineral (milligrams per kilogram of diet): Mn, 100; Zn, 70; Fe, 50; Cu, 10; iodine, 1; Se, 0.30; antioxidant, 50.

**Table 2:** Bioactive phytochemical compound content and amino acid profile of amaranth grain (*Amaranthus hybridus chlorostachys*).

Item	Value
ß-Sitosterol (mg/kg)	1197.53
Stigmasterol (mg/kg)	609.46
Δ-5-Avena sterol (mg/kg)	253.2
Campesterol (mg/kg)	808.79
Δ-7-Stigmastanol (mg/kg)	276.62
Total phytosterols (mg/kg)	3194.28
α-Tocopherol (ppm)	18.60
Δ- Tocopherol (ppm)	219.07
β and γ-Tocopherols (ppm)	293.18
Total tocopherol (ppm)	530.85
EE (g/kg)	52
α-Linoleic acid (C18:2, n-6) (g/kg)	347.9
Squalene (ppm)	2161.39
Crude protein (g/kg)	168
Arginine	7.1
Histidine	2.3
Isoleucine	3.3
Leucine	6.2
Lysine	5.4
Methionine	2.8
Phenylalanine	4.3
Threonine	3.8
Tryptophan	1.5
Valine	4.2
Alanine	4.1
Aspartic acid	10.0
Cysteine	2.1
Glutamic acid	16.1
Glycine	7.2
Proline	3.6
Tyrosine	4.0
Serine	6.1
Non- aa nitrogen	73.9
Sum of EAA	40.9
Sum of NEAA	53.2

**Table 3:** Main effects of amaranth feeding, enzyme additionand interaction between amaranth feeding and enzyme addition on productive performance of laying hens.

Item	FI ^1^ (g d^−1^ Bird^−1^)	HDP ^2^ (%)	EW ^3^ (g)	EM ^4^ (g d^−1^ Bird^−1^)	FCR ^5^ (kg Feed: kg Egg)
Amaranth Grain (AG), (g/kg)					
0	109.17	81.80	60.56 ^a^	49.54 ^a^	2.20 ^b^
100	109.08	82.25	59.80 ^a^	49.20 ^a^	2.21 ^b^
200	109.13	82.36	59.64 ^a^	49.13 ^a^	2.22 ^b^
300	108.11	80.13	58.24 ^b^	46.68 ^b^	2.33 ^a^
400	108.97	79.12	58.49 ^b^	46.29 ^b^	2.35 ^a^
SEM	0.131	0.277	0.319	0.352	0.016
*p*-value	0.85	0.47	0.0001	0.0001	0.0001
*p*-value					
Contrasts	0.49	0.01	0.001	0.001	0.001
Linear	0.37	0.001	0.001	0.001	0.001
Quadratic	0.76	0.001	0.53	0.06	0.07
Enzyme (±E), (g/kg)					
0 (−E)	109.18	80.13	58.90 ^b^	47.20 ^b^	2.31 ^a^
0.25 (+E)	109.00	82.14	59.80 ^a^	49.13 ^a^	2.22 ^b^
SEM	0.082	0.175	0.202	0.222	0.010
*p*-value	0.13	0.36	0.003	0.0001	0.0001
Interactions effect, (AG × E)					
T_1_: 0 × 0	109.03	81.45	60.20 ^ab^	49.04 ^bc^	2.22 ^cd^
T_2_: 100 × 0	109.37	80.58	59.56 ^b^	48.00 ^cd^	2.28 ^bc^
T_3_: 200 × 0	109.38	80.13	59.24 ^bc^	47.47 ^cd^	2.30 ^b^
T_4_: 300 × 0	109.13	79.68	57.49 ^d^	45.81 ^e^	2.38 ^a^
T_5_: 400 × 0	109.01	78.79	57.99 ^cd^	45.69 ^e^	2.39 ^a^
T_6_: 0 × 0.25	109.31	82.14	60.91 ^a^	50.03 ^ab^	2.18 ^de^
T_7_: 100 × 0.25	108.78	83.92	60.05 ^ab^	50.40 ^ab^	2.15 ^de^
T_8_: 200 × 0.25	109.22	84.59	60.04 ^ab^	50.80 ^a^	2.14 ^e^
T_9_: 300 × 0.25	108.88	80.58	58.89 ^bc^	47.54 ^cd^	2.29 ^bc^
T_10_: 400 × 0.25	108.93	79.46	58.99 ^bc^	46.88 ^de^	2.32 ^ab^
SEM	0.185	0.392	0.452	0.497	0.023
*p*-value	0.15	0.64	0.0001	0.0001	0.0001

^a–e^ Means within each column with different superscripts differ (*p* < 0.05). **T_1_**: 0 AG-E, **T_2_**: 100 AG-E, **T_3_**: 200 AG-E, **T_4_**: 300 AG-E, **T_5_**: 400 AG-E, **T_6_**: 0 AG + E, **T_7_**: 100 AG + E, **T_8_**: 200 AG + E, **T_9_**: 300 AG + E, **T_10_**: 400 AG + E; ^1^ **FI**: feed intake, ^2^ **HDP**: hen-day production, ^3^ **EW**: egg weight, ^4^ **EM**: egg mass, ^5^ **FCR**: feed conversion ratio.

**Table 4:** Main effects of amaranth feeding, enzyme additionand interaction between amaranth feeding and enzyme addition on egg quality traits of laying hens.

Item	Shell Thickness (mm)	Shell Strength (kg/cm^2^)	Shape Index (%)	Egg Specific Gravity (g cm^−3^)	Haugh Unit	Yolk Cholesterol (mg/g yolk)	Egg Cholesterol (mg/egg yolk)
Amaranth Grain (AG), (g/kg)							
0	0.306	3.323	75.04	1.083	79.66	12.59 ^a^	218.26 ^a^
100	0.313	3.350	75.17	1.085	80.76	11.78 ^ab^	204.94 ^ab^
200	0.311	3.343	75.04	1.084	80.67	11.74 ^ab^	203.68 ^ab^
300	0.313	3.350	74.88	1.084	80.36	11.33 ^b^	195.02 ^b^
400	0.303	3.355	74.82	1.083	80.17	11.30 ^b^	194.87 ^b^
SEM	0.005	0.025	0.919	0.001	0.666	0.329	5.730
*p*-value	0.85	0.99	0.99	0.95	0.78	0.04	0.04
*p*-value							
Contrasts	0.84	0.38	0.95	0.48	0.27	0.001	0.04
Linear	0.83	0.45	0.80	0.71	0.77	0.001	0.001
Quadratic	0.85	0.76	0.91	0.73	0.26	0.34	0.38
Enzyme (±E), (g/kg)							
0 (−E)	0.310	3.335	75.00	1.083	80.40	11.81	203.98
0.25 (+E)	0.311	3.354	74.99	1.085	80.25	11.70	202.73
SEM	0.003	0.016	0.581	0.001	0.421	0.208	3.624
*p*-value	0.83	0.42	0.99	0.33	0.80	0.72	0.81
Interactions effect (AG × E)							
T_1_: 0 × 0	0.307	3.317	75.10	1.083	79.73	12.77	220.99
T_2_: 100 × 0	0.311	3.335	75.18	1.085	80.61	11.81	205.15
T_3_: 200 × 0	0.307	3.337	74.83	1.083	80.56	11.79	203.75
T_4_: 300 × 0	0.310	3.340	74.86	1.083	80.82	11.32	194.56
T_5_: 400 × 0	0.315	3.345	75.00	1.083	80.29	11.34	195.46
T_6_: 0 × 0.25	0.305	3.330	74.98	1.085	79.58	12.42	215.53
T_7_: 100 × 0.25	0.317	3.365	75.16	1.085	80.92	11.76	204.74
T_8_: 200 × 0.25	0.315	3.350	75.25	1.087	80.78	11.70	203.61
T_9_: 300 × 0.25	0.307	3.360	74.90	1.084	79.90	11.34	195.48
T_10_: 400 × 0.25	0.310	3.365	74.64	1.086	80.06	11.27	194.28
SEM	0.007	0.036	1.300	0.002	0.942	0.466	8.104
*p*-value	0.85	0.99	0.99	0.90	0.97	0.99	0.99

^a^,^b^ Means within each column with different superscripts differ (*p* < 0.05). **T_1_**: 0 AG-E, **T_2_**: 100 AG-E, **T_3_**: 200 AG-E, **T_4_**: 300 AG-E, **T_5_**: 400 AG-E, **T_6_**: 0 AG + E, **T_7_**: 100 AG + E, **T_8_**: 200 AG + E, **T_9_**: 300 AG + E, **T_10_**: 400 AG + E.

**Table 5:** Main effect of amaranth feeding, enzyme addition and interaction between amaranth feeding and enzyme addition on serum biochemical parameters of laying hens.

	Antioxidant Indices Plasma				Lipid Profile Plasma (mg dL^−1^)				
Items	TAC ^1^ (U mL^−1^)	MDA ^2^ (nmol mL ^−1^)	ALT ^3^ (U L^−1^)	AST ^4^ (U L^−1^)	TG ^5^	TC ^6^	LDL ^7^	HDL ^8^	AI ^9^
Amaranth Grain (AG), (g/kg)									
0	6.20 ^b^	4.84	5.30 ^a^	215.6 ^a^	96.39 ^a^	105.86 ^a^	97.80 ^a^	40.38 ^b^	2.42 ^a^
100	6.44 ^a^	5.24	4.82 ^ab^	213.2 ^ab^	92.36 ^b^	98.41 ^b^	95.55 ^b^	49.87 ^a^	2.23 ^b^
200	6.49 ^a^	5.26	4.69 ^ab^	212.0 ^b^	91.52 ^b^	97.55 ^bc^	95.69 ^b^	50.22 ^a^	2.24 ^b^
300	6.46 ^a^	5.03	4.65 ^b^	213.3 ^ab^	90.96 ^b^	96.45 ^c^	94.57 ^b^	51.68 ^a^	2.18 ^c^
400	6.49 ^a^	4.99	4.69 ^ab^	213.0 ^ab^	90.33 ^b^	96.10 ^c^	94.66 ^b^	50.50 ^a^	2.40 ^a^
SEM	0.076	0.204	0.205	1.112	1.190	0.655	0.565	0.626	0.010
*p*-value	0.04	0.58	0.03	0.05	0.01	0.0001	0.002	0.0001	0.0001
*p*-value									
Contrasts	0.001	0.22	0.01	0.03	0.001	0.001	0.001	0.001	0.001
Linear	0.01	0.89	0.04	0.15	0.001	0.001	0.001	0.001	0.001
Quadratic	0.08	0.16	0.15	0.11	0.12	0.001	0.11	0.001	0.001
Enzyme (±E), (g/kg)									
0 (−E)	6.39	5.08	4.87	213.61	91.83	99.52	95.85	48.19	2.23 ^b^
0.25 (+E)	6.44	5.06	4.79	213.31	92.80	98.23	95.45	48.87	2.35 ^a^
SEM	0.048	0.129	0.130	0.703	0.752	0.414	0.357	0.396	0.0001
*p*-value	0.49	0.99	0.68	0.77	0.37	0.36	0.44	0.24	0.004
Interactions effect (AG × E)									
T_1_: 0 × 0	6.17	4.79	5.45	216.36	93.06	106.73 ^a^	98.55 ^a^	40.23 ^b^	2.45 ^b^
T_2_: 100 × 0	6.43	5.65	4.90	213.02	92.58	99.18 ^b^	95.70 ^b^	49.42 ^a^	2.18 ^e^
T_3_: 200 × 0	6.48	5.18	4.71	211.95	91.33	98.58 ^bc^	95.73 ^b^	50.22 ^a^	2.19 ^e^
T_4_: 300 × 0	6.43	4.91	4.67	213.62	91.44	97.24 ^bcd^	94.49 ^b^	51.64 ^a^	2.07 ^f^
T_5_: 400 × 0	6.46	4.88	4.61	213.10	90.73	95.87 ^cd^	94.80 ^b^	50.45 ^a^	2.29 ^d^
T_6_: 0 × 0.25	6.23	4.90	5.15	214.89	99.73	104.98 ^a^	97.05 ^ab^	40.53 ^b^	2.39 ^c^
T_7_: 100 × 0.25	6.46	4.83	4.74	213.55	92.14	97.65 ^bcd^	95.70 ^b^	50.33 ^a^	2.29 ^d^
T_8_: 200 × 0.25	6.50	5.34	4.67	212.05	91.72	96.52 ^bcd^	95.65 ^b^	50.22 ^a^	2.30 ^d^
T_9_: 300 × 0.25	6.49	5.16	4.63	213.10	90.48	95.67 ^d^	94.65 ^b^	51.72 ^a^	2.30 ^d^
T_10_: 400 × 0.25	6.53	5.10	4.77	212.97	89.93	95.87 ^cd^	94.53 ^b^	51.56 ^a^	2.50 ^a^
SEM	0.107	0.289	0.290	1.573	1.682	1.161	0.800	0.886	0.014
*p*-value	0.99	0.32	0.95	0.98	0.15	0.02	0.04	0.04	0.001

^a–f^ Means within each column with different superscripts differ (*p* < 0.05). **T_1_**: 0 AG-E, **T_2_**: 100 AG-E, **T_3_**: 200 AG-E, **T_4_**: 300 AG-E, **T_5_**: 400 AG-E, **T_6_**: 0 AG + E, **T_7_**: 100 AG + E, **T_8_**: 200 AG + E, **T_9_**: 300 AG + E, **T_10_**: 400 AG + E; ^1^ **TAC**: total antioxidant capacity, ^2^ **MDA**: malondialdehyde, ^3^ **ALT**: alanine aminotransferase, ^4^ **AST**: aspartate aminotransferase, ^5^ **TG**: triglyceride, ^6^ **TC**: total cholesterol, ^7^ **LDL**: low-density lipoprotein, ^8^ **HDL**: high-density lipoprotein, ^9^ **AI**: atherogenic index (LDL/HDL).

## Data Availability

The data from this study are available from the corresponding author upon reasonable request.
